# The Adsorption of Ru-Based Dyes on the TiO_2_ Surface to Enhance the Photovoltaic Efficiency of Dye-Sensitized Solar Cell Devices

**DOI:** 10.3390/molecules30061312

**Published:** 2025-03-14

**Authors:** Malgorzata Makowska-Janusik, Katarzyna Filipecka-Szymczyk, Daniel Pelczarski, Waldemar Stampor, Maciej Zalas

**Affiliations:** 1 Faculty of Science and Technology, Jan Dlugosz University, Al. Armii Krajowej 13/15, 42-200 Czestochowa, Poland; k.filipecka-szymczyk@ujd.edu.pl; 2 Department of Molecular Photophysics, Institute of Applied Physics and Mathematics, Gdansk University of Technology, 11/12 Narutowicza str., 80-233 Gdansk, Poland; daniel.pelczarski@pg.edu.pl (D.P.); waldemar.stampor@pg.edu.pl (W.S.); 3 Faculty of Chemistry, Adam Mickiewicz University, 8 Uniwersytetu Poznańskiego str., 61-614 Poznan, Poland; maciej.zalas@amu.edu.pl

**Keywords:** DSSC, dyes, adsorption, titanium dioxide, Monte Carlo, tris(bipyridine) ruthenium(II) complexes

## Abstract

Adsorption of mononuclear tris(bipyridine) ruthenium(II) complexes and binuclear tris(bipyridine) ruthenium(II) complexes equipped with carboxyl groups (-COOH) on the (111) surface of TiO_2_ crystal in anatase form was modeled using Monte Carlo simulations, applying the Universal force field. It was shown that the adsorption efficiency of the ruthenium-based dyes on the TiO_2_ surface depends on the position of the anchoring -COOH group in the molecular structure. The increase in the number of possible anchor groups in the dyes increases their ability to deposit on the surface of semiconductors. The chemisorbed molecules, such as mononuclear tris(bipyridine) ruthenium(II) complexes with the -COOH group in para position (RuLp) and binuclear tris(bipyridine) ruthenium(II) complexes called B3 with two anchoring -COOH groups and phenyl in the spacer, interact with the adsorber and other neighboring dyes, changing their electron and optical properties. The obtained computational results help to explain the behavior of the dyes on the TiO_2_ surface, giving impact on their DSSC applications.

## 1. Introduction

Energy drives the development of the world, and its demand increases with technological progress. Excessive use of fossil fuels leads to their depletion and environmental pollution. The solution is renewable energy, obtained from natural sources such as the sun, wind, or water. Sunlight can be converted into electrical energy in a photovoltaic (PV) process using solar cells. Dye-sensitized solar cells (DSSCs) are considered one of the most promising PV technologies [1,2]. They represent an alternative to traditional silicon-based photovoltaic technologies, primarily due to their low cost, ease of fabrication, and flexibility [3,4]. Additionally, DSSCs are more environmentally friendly than traditional solar cells. The photoconversion efficiency of DSSCs has progressed from 7.1% [5] to 15.2% [6], and their development is constantly in progress. In DSSCs, organic dye molecules are adsorbed on the surface of a wide-band-gap semiconductor, which absorb light solely in the ultraviolet region. The role of dyes is to harvest solar light and give rise to charge separation by injecting electrons into the conduction band of the semiconductor. A critical aspect of DSSC performance lies in the interaction between dye molecules and the semiconductor surface, typically titanium dioxide (TiO_2_) [7]. This interaction is fundamental to the efficiency of charge generation and its transport within the cell.

As DSSCs were developed, a large number of dyes with improved molar absorption coefficients in visible light and an appropriate arrangement of the frontier orbitals to the used semiconductor were synthesized [8]. However, to obtain high quantum yields of the electron transfer process from the dye to the semiconductor, the dye needs to be in contact with the semiconductor. Moreover, the dye must be able to strongly adsorb onto the semiconductor surface to efficiently inject electrons into the semiconductor. The interaction between dye molecules and the TiO_2_ surface is influenced by several factors, including the chemical structure of the dye, the nature of the anchoring groups, and the surface properties of the semiconductor. Dye molecules must contain a chemical substituent that can anchor the chromophore onto the metal oxide via adsorption. Various anchoring groups have been successfully demonstrated in DSSC devices, e.g., carboxylate, phosphonate, sulfonate, salicylate, 8-hydroxyl quinoline, acetylacetonate, catechol, hydroxyl, pyridyl, and hydroxamate [9,10,11,12]. Dyes designed with functional groups such as carboxylic acids (-COOH) facilitate strong adsorption onto the TiO_2_ surface [12]. However, they coordinate in different ways to metal ions: simply by physical adsorption, via hydrogen bonding or chemical bond formation with an unidentate linkage, a bidentate (or chelating) linkage, or a bridging linkage ensuring a stable attachment essential for effective electron transfer [13].

Dyes anchoring at the surface of semiconductors have been investigated using different experimental [14,15] and computational techniques [16,17,18]. Along with experimental studies, computer simulations have contributed to a deeper understanding of the electronic structure and optical properties of new dyes and to unraveling the dye–semiconductor interactions characterizing the crucial DSC heterointerface. The study of the adsorption mechanism of dye molecules on the surfaces of adsorbent material can explain the dye behaviors at the surface of the defined semiconductor. In this case, molecular dynamics (MD) simulation is an important computational method that provides significant quantitative and qualitative information on the interactions involved in the adsorption process of organic molecules. MD and Monte Carlo simulations were employed to investigate the adsorption of methylene blue and rhodamine B on graphene and graphene oxide [19]. Density Functional Theory (DFT) was used to model the adsorption of TPA-based dyes on TiO_2_, revealing different adsorption modes and electronic coupling mechanisms [20]. A combination of statistical physics modeling and DFT was applied to study the adsorption of paprika dye on TiO_2_, identifying multiple binding modes [18]. An integrated approach using FT-IR measurements and DFT calculations was used to determine the most favorable adsorption modes for organic dyes on TiO_2_, with implications for DSSC performance [17]. In the series of Angelis and coworkers’ papers, the authors investigated the adsorption of small dyes on the nanostructure of TiO_2_, applying DFT methods of calculations [17,20]. The adsorption of formic acid on the stoichiometric anatase (101) surface has been studied using DFT calculations with a TiO_2_ slab geometry [21]. The above-mentioned works report the modeling of small (low-electron-content) molecules to save time and the computational power of modeling. To our best knowledge, the adsorption of metal–organic molecules, popular for use as dyes in DSSC applications, has never been investigated computationally using statistical methods. In our study, we propose the crystal approach with three-dimensional boundary conditions to model adsorption, taking into consideration the intermolecular interaction at real working temperature. In this case, by minimizing the total energy of the composite, the dyes look for the appropriate sites, providing a composite structure in its thermodynamic equilibrium state. This approach closely reflects the dynamic and structural properties of the real material. The performed studies demonstrate the importance of computational modeling in understanding dye–semiconductor interactions for various applications.

**Figure 1:**
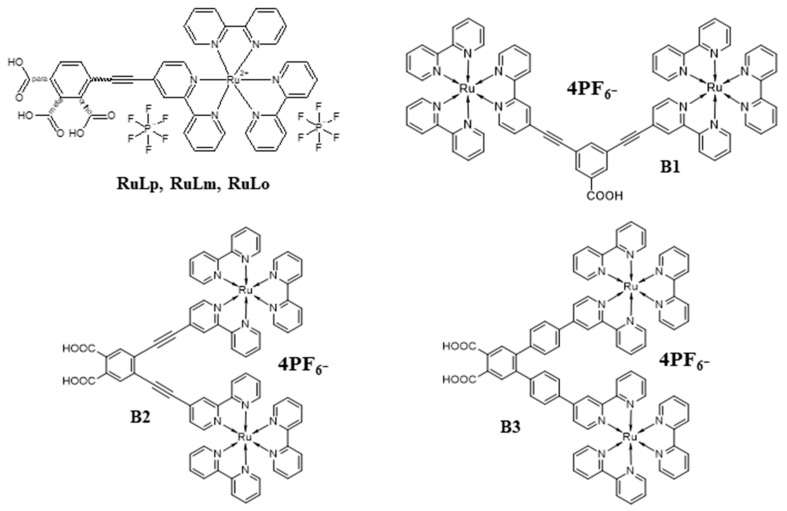
Structures of the investigated dye molecules, mononuclear tris(bipyridine) ruthenium(II) complexes (RuLm, RuLo, and RuLp) and binuclear tris(bipyridine) ruthenium(II) complexes (**B1**, **B2**, and **B3**).

In the presented work, we focus on investigations of the adsorption mechanism of mononuclear tris(bipyridine) ruthenium(II) complexes (RuLp, RuLo, and RuLm) [22] and binuclear tris(bipyridine) ruthenium(II) complexes BX (B1, B2, and B3) [23] equipped with carboxyl groups (-COOH), presented in Figure 1, at the (111) surface of TiO_2_ in anatase form. The B2 and B3 molecules possess two anchor groups to improve their adsorption ability and binding strength, which are known factors for improving electron transport and electron injection in DSSCs [24]. The photophysical properties of the RuLp, RuLo, and RuLm molecules and their efficiency as the dyes in DSSCs were reported in our previous work [22]. Their measured UV–vis absorption spectra are quite similar to one other, with three major absorption bands at 245, 290, and 330 nm and a broader one, in the visible region, between 400 and 520 nm, important in the absorption of sunlight in photovoltaic devices. The absorption peak located in the visible range is assigned to metal-to-ligand charge transfer (MLCT) transitions. The molar extinction coefficient of these three molecules in the visible range is at the same level, equal to 2.0 × 10^4^ M^−1^ cm^−1^. The BX molecules also exhibit a broad UV–vis absorption band, with maxima at 460 nm corresponding to the MLCT transition, characteristic of ruthenium polypyridine complexes [23]. The molar extinction coefficient at the MLCT band changes as follows, B3 > B1 > B2, possessing the values 4.2, 4.1, and 3.7 × 10^4^ M^−1^ cm^−1^, respectively. It should be mentioned that the investigated molecules were synthesized not to reach the DSSC efficiency record but to try to find the answer to the question of the effect of the position and quantity of the anchoring group on the overall photon-to-current efficiency of the DSSC.

The presented studies, which report the adsorption of the dyes on the TiO_2_ surface, were performed using Monte Carlo simulations based on statistical–mechanical methods. Our previous research shows that the -COOH anchor position in the dye molecule plays an important role in the electron injection process and also the deposition of the dyes on the surface of the semiconducting electrode [22]. There are still a limited number of studies based on computer simulation in the investigation of the mechanism of dye molecule adsorption characteristics on TiO_2_.

## 2. Results

The (111) surface of the TiO_2_ crystal structure in anatase form was constructed. The (111) surface of TiO_2_ is preferred for dye adsorption in DSSCs due to its superior properties. In the work of Amoli et al., it was reported that the (111) surface of TiO_2_ was selected to adsorb dyes in DSSC applications due to its high dye adsorption and fast electron transport dynamics compared to other crystal facets [25]. DSSCs fabricated with TiO_2_ nanostructures exposing (111) facets show higher power conversion efficiency (9.60%) compared to those with (101) facets (7.59%). The chosen surface provided the basis for constructing a unit cell with edges equal to 37.94 Å × 21.36 Å × 66.68 Å containing the semiconductor and organic dyes. To ensure the studied unit cells had the same volume for all modeled dyes, three mono-ruthenium molecules (RuLp, RuLm, and RuLo) or two of the bi-ruthenium molecules (BX = B1, B2, and B3) were located in the orthorhombic unit cell. To simulate the dye adsorption on the surface of the TiO_2_, the hydrogen atom from the -COOH of the RuLp, RuLo, RuLm, and B1 molecules was removed. Concerning B2 and B3 molecules, two possibilities were taken into consideration. In the first case, a hydrogen atom was removed from one carboxyl group (these molecules are named B2_H_ and B3_H_). In the second case, the hydrogens from both existing carboxyl groups were deleted. This approach allows us to discuss the possibility of attaching the dye using one or two dye–semiconductor connections. The idea to remove hydrogen atoms creating bonds between the dye and TiO_2_ was created as a result of literature reports. The -COOH group typically anchors to TiO_2_ via bidentate chelating and ester bonds, with the -COO- group deprotonating upon adsorption [26]. This anchoring mode is energetically favored and results in strong electronic coupling between the dye and TiO_2_, potentially leading to adiabatic electron injection [20].

The electrostatic interactions between dyes and the surface of the semiconductor were calculated by applying the atom charges coming from quantum chemical calculations with computational parameters described in Section 4. The probabilistic behavior of the organic dyes at the surface of the TiO_2_ was modeled using Monte Carlo simulations based on molecular mechanic methods implemented in the Materials Studio package. In the performed simulations, the Universal force field was used to simulate the most probable location of the organic dyes on the (111) TiO_2_ anatase structure.

**Figure 2:**
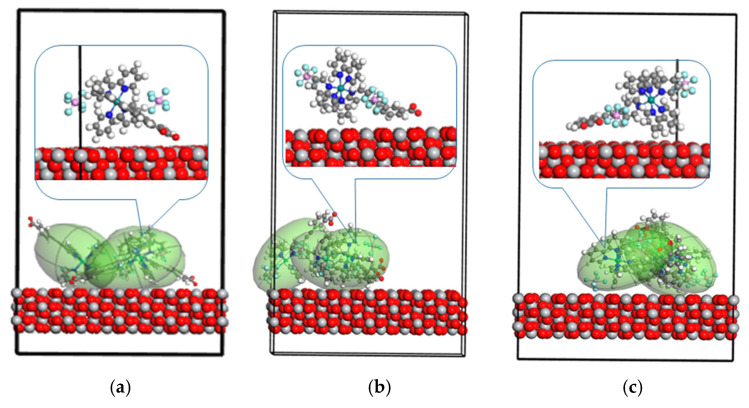
The composites obtained by the Monte Carlo simulations, based on the (111) surface of the TiO_2_ anatase structures and RuLp (**a**), RuLm (**b**), and RyLo (**c**) molecules.

**Figure 3:**
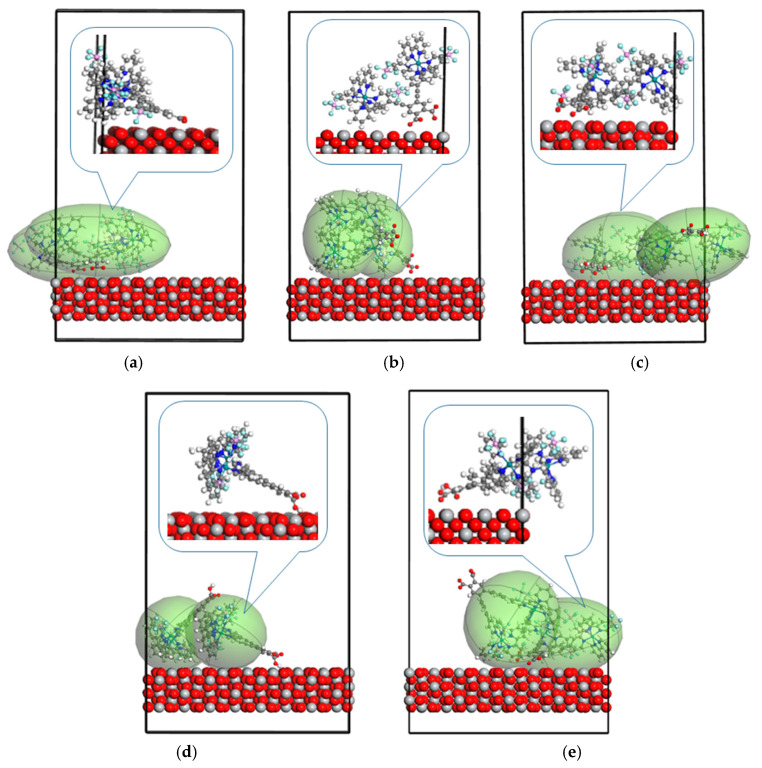
The composites obtained by the Monte Carlo simulations based on the (111) surface of the TiO_2_ anatase structures and B1 (**a**), B2 (**b**), B3 (**c**), B2_H_ (**d**), and B2_H_ (**e**) without any hydrogen on the anchor group (**a**–**c**) and with one hydrogen left on the anchor group (**d**,**e**).

As the output of the performed Monte Carlo simulations, the structures presented in Figure 2 and Figure 3 were obtained. They show the energetically favorable configurations of the dyes and their adsorption at the suitable sites of the (111) TiO_2_ surface. The Monte Carlo method was used to calculate the adsorption parameters and binding energy of dye/TiO_2_ composites. Titanium atoms on the TiO_2_ surface were proposed to act as active sites for anchoring the dye molecules. The interaction occurring between -COOH groups of the Ru-bipyridine complexes with the TiO_2_ surface was expected to result in effective monolayer anchoring of the dye molecules onto TiO_2_ films. The created dye–TiO_2_ linkages should form an electronic coupling between the cationic metal surface site (Ti) and the oxygen of the carboxyl group. The carboxyl (-COOH) group anchors to TiO_2_ surfaces via the Ti atoms. The interaction occurs through the oxygen atoms in the carboxyl group, forming coordinate bonds with the titanium atoms on the TiO_2_ surface. Studies show that the carboxylic group deprotonates, and the resulting COO- anchors to TiO_2_ via multiple modes, including bidentate chelating and ester bonds [26]. The adsorption geometry and strength can be influenced by spacer groups between the dye and the anchor, affecting electron transfer rates across the dye–TiO_2_ interface [27].

**Figure 4:**
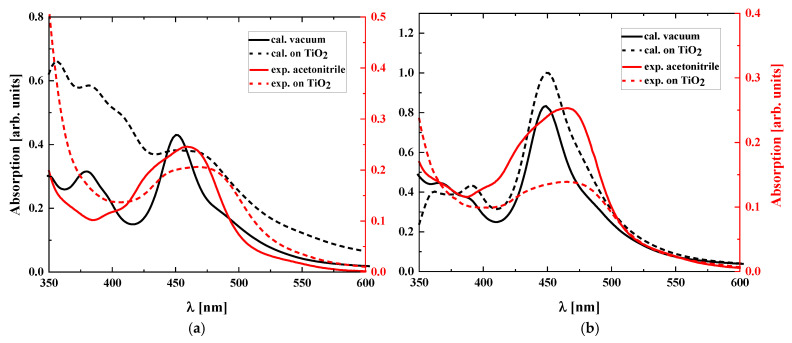

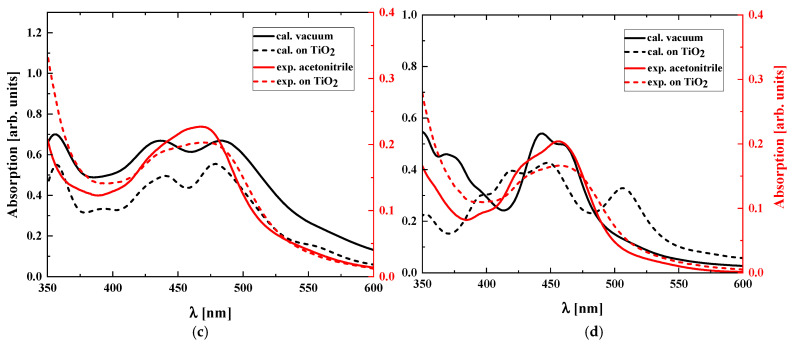
UV–vis absorption spectra calculated using DFT/B3LYP method for RuLp (**a**), B1 (**b**), B2 (**c**), and B3 (**d**) molecules in vacuum or deposited on TiO_2_ (111) surface and measured experimentally in acetonitrile and deposited on TiO_2_ thin film.

The modeled dye/TiO_2_ structures, presented in Figure 2 and Figure 3, were used to investigate the environmental effect on the optical properties of the studied dyes and possible reactions between the dyes and semiconductor. The dye/TiO_2_ systems with RuLp, B1, B2, and B3 molecules were chosen as the structures with probable chemisorption occurring at the (111) surface of the TiO_2_. Based on the discrete local field model [28], the UV–vis spectra of the RuLp, B1, B2, and B3 adsorbed on the TiO_2_ surface were calculated. Also, the UV–vis absorption spectra of these molecules in a vacuum were modeled and the results are presented in Figure 4. The computed data are compared with the experimental UV–vis spectra measured in acetonitrile as a solvent and for the molecules deposited on the thin film of the TiO_2_. We focused on the long-wavelength part of the spectra because electronic transitions in this region are assigned to MLCT absorption and have a decisive influence on the absorption of sunlight by the dye.

## 3. Discussion

The used adsorption locator module and Monte Caro simulations, implemented in the Materials Studio (version: MS 8.0) program package, give the possibility to calculate the adsorption configurations and binding energy of the studied composites. The corresponding energies of the investigated structures are listed in Table 1. The total energy of the configurations obtained includes the energies of adsorbate components, deformation energy upon adsorption, and rigid adsorption energy. One can see that the highest stability is presented by the composite of RuLp dyes adsorbed on the (111) surface of the TiO_2_, possessing a total energy per atom equal to 0.45 × 10^3^ kcal/mol. The other mononuclear tris(bipyridine) ruthenium(II) complexes, such as RuLm/TiO_2_ and RuLo/TiO_2_, compose less stable composites, even compared to the binuclear tris(bipyridine) ruthenium(II) complexes. All ruthenium-based binuclear dyes on the TiO_2_ surface possess similar stability.

The adsorption energy (*E_ads_*) is the energy change when a molecule adsorbs onto the surface of the semiconductor. It helps identify the most stable adsorption sites of the dyes and is calculated by the following formula:

(1) $$E_{ads}=E_{tot}-\left(E_{surface}+E_{adsorbate}\right),$$

where *E_tot_* is the total energy of the composite system, *E_surface_* is the energy of the clean surface, and *E_absorbate_* is the energy of an isolated adsorbed molecule. The calculated *E_ads_* is negative for all investigated composites. This indicates exothermic adsorption, which means favorable binding. The strongest interaction is observed between RuLp or RuLm molecules and the TiO_2_ surface. It is also seen that the mononuclear tris(bipyridine) ruthenium(II) complexes interact with the (111) surface of the TiO_2_ more strongly than the binuclear tris(bipyridine) ruthenium(II) complexes. The reaction of the binuclear complexes is the most significant for the B2_H_, B3, and B3_H_ molecules, indicating that the adsorption of the B2 molecules engages mostly one -COOH group. The adsorption of B3 does not have these limitations; even more favorable is the connection of the dye and TiO_2_ via two -COOH groups.

$\partial E_{ads}/\partial N_{i}$ means the change in adsorption energy when one molecule is released from the adsorbing surface. For all molecules considered, the energy change is negative, which means that removing one molecule increases the adsorption energy for the remaining molecules. The most important change is seen for the RuLp molecules. It can be concluded that the adsorbed RuLp molecules interact with each other in a repulsive manner, and removing one of them causes the subsequent molecules to adsorb more strongly. In Figure 2a, it is seen that the RuLp molecules tend to arrange their dipole moment perpendicularly to the adsorber surface. Unfortunately, their size does not allow such an arrangement of all molecules at the same time. Removing one of them would make it possible to reduce the repulsive interaction between the molecules and promote the adsorption of all the remaining molecules in the form of a monolayer. The desorption mechanism, in this case, will probably follow the model of sequential, hindered desorption, where the first molecules desorb more easily, but each subsequent one is more closely bound to the surface. The desorption of the molecules RuLo or RuLm is much easier than is observed for the RuLp molecules. This is in agreement with our previous experimental studies, which showed that substitution of the anchor group in the meta position of the mononuclear tris(bipyridine) ruthenium(II) complexes weakens the connectivity between the molecule and TiO_2_, while the ortho position causes an even greater decrease in the adsorption capacity of the RuLo dye due to steric hindrance occurring between the -COOH group and the remaining components of the dye molecule [22]. Such behavior of RuLm and RuLo molecules causes a weakening of the charge transfer occurring between the dye and the semiconductor in their DSSC applications.

Unfavorable intermolecular interactions that do not allow BX molecules to settle on the TiO_2_ surface as a monolayer are also visible for structures B2 and B3 (see Figure 3). The significant $\partial E_{ads}/\partial N_{i}$ energy changes observed for B2, B2_H_, and B3 give the information that removing one molecule from the surface of the TiO_2_ increases the dye–semiconductor interaction. The high value of the $\partial E_{ads}/\partial N_{i}$ for the B2 molecule may be due to the aggregation of the dye on the semiconductor surface, which we experimentally found in our previous work [23]. According to Funaki et al. [29], the aggregation phenomenon of the dye is more likely the more phenylene–ethylene groups there are in its structure, as is the fwithB2.

The adsorbate-to-surface distance (see Table 2) can help us to understand the nature of the molecule’s location at the surface of the semiconductor. RuLp, B1, and B2, as well as the B3, are connected to the TiO_2_ surface via chemisorption. This can be argued because the distance between the -COOH group and the TiO_2_ surface is much less than 2 Å. This allows us to conclude that the B2 and B3 molecules prefer a bidentate connection with the semiconductor. The RuLm and RuLo molecules are probably connected with the semiconductors via physisorption. These results are in agreement with our previous experimental studies, where we show that the introduction of two anchor groups in dyes B2 and B3 results in a significant improvement in adsorption capacity compared to B1 [23]. The presence of multiple anchoring sites increases the likelihood of effective adsorption onto the TiO_2_ surface, which is crucial for improving electron transport and charge injection in DSSCs. The calculations show that for the organic dyes possessing a -COOH as an anchoring group, the preferred adsorption mode is bidentate bridging, with one proton transferred to nearby surface oxygen, while monodentate anchoring is usually predicted to be less stable [20].

The UV–vis absorption spectra of the RuLp, B1, B2, and B3 molecules chemisorbed on the surface of TiO_2_ are also discussed. The experimental measurements were performed for the mentioned molecules deposited on the semiconductor and solved in acetonitrile. The obtained data were compared with the computational results obtained for the isolated molecules and the ones affected by the local field mimicking the environmental effect. The investigated Ru complexes have a broad absorption band in the spectral range 400–500 nm [30,31] (see Figure 4). The maxima of the MLCT bands measured in acetonitrile for B1 and B2 dyes show a bathochromic shift with respect to B3 dye. This is related to the structure of the anchoring ligands and can be explained by the fact that resonance-stabilized aromatic phenyls cause a reduction in the electron-withdrawing properties of B3 dye. The introduction of a second electron-withdrawing group (-COOH) into the dye structure reduces its absorption properties [32], while the introduction of phenyl groups increases the value of the molar absorption coefficient [29]. The deposition of the investigated molecules on the surface of TiO_2_ does not significantly change the position of the MLCT band. In all cases, the intermolecular interaction flattens the peak by reducing the absorption intensity. The performed DFT/B3LYP calculations can reproduce the MLCT absorption, taking into consideration its spectral position. Analyzing the UV–vis absorption spectra calculated for the B2 molecule in a vacuum and on the TiO_2_ surface, one can conclude that both spectra (see Figure 4b) are very similar. This means that the occurring intermolecular interaction between molecules is almost negligible. For the RuLp molecule, the spectrum obtained for the dye deposited on the TiO_2_ is more broad, and its maximum is shifted into the longwave direction, which can be caused by the chemical bonds occurring between the dye and semiconductor. The most visible changes are seen in spectra calculated for the B3 molecule. This confirms the existence of electronic coupling between the frontier orbitals of the dye and the conduction band of the semiconductor, which reduces the energy difference of the electronic transition. This can be concluded by observing the shift of the absorption peak towards the red side of the spectrum (see Figure 4d). The reaction between B3 dye and semiconductor is visible.

**Figure 5:**
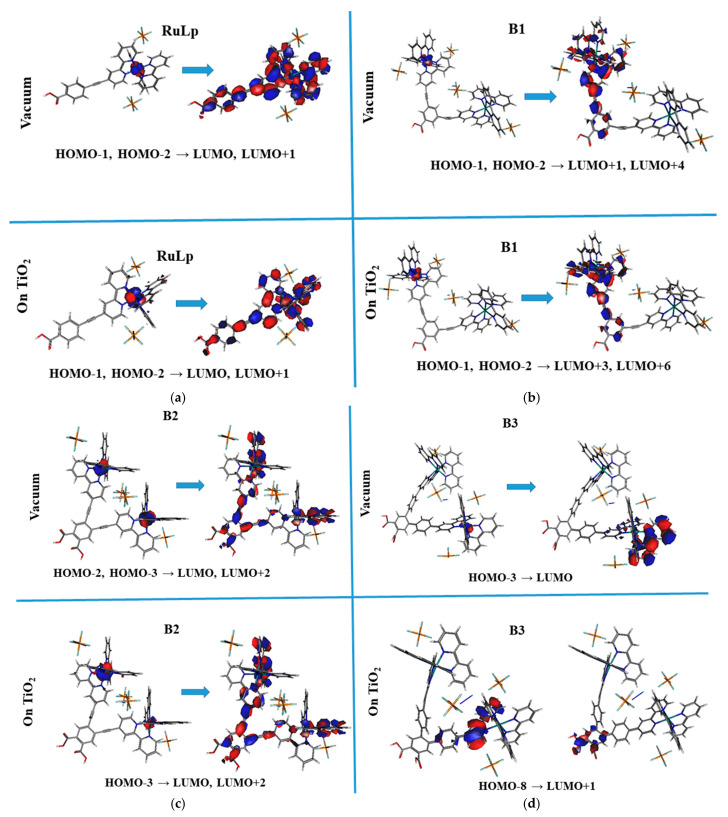
The molecular orbitals involved in electron transition, causing the UV–vis absorption band in the MLCT region with the highest intensity, calculated using the DFT/BELP method for the isolated RuLp (**a**), B1 (**b**), B2 (**c**), and B3 (**d**) molecules and those deposited on TiO_2_ (111) surface.

The molecular orbitals involved in electron transition, causing the UV–vis absorption band in the MLCT region with the highest intensity, presented in Figure 4, calculated using the DFT/B3LYP method for the isolated RuLp, B1, B2, and B3 molecules and those deposited on the TiO_2_ (111) surface are presented in Figure 5. One may see that the ground molecular state is located at the Ru atom. The excited state is more extended on the spacer, located between the anchor group and the bipyridine complex. The local field, which models the dye–environment interaction, does not significantly affect the ground and excited orbitals. One exception is seen in the case of the B3 molecule. Its excited state is located at the anchor group, which enables the charge transfer between the dye molecule and the semiconductor. This conclusion is consistent with our previous studies presented in the paper [33].

## 4. Computer Simulation Methodology

### 4.1. Computer Simulations

The presented work concerns quantum chemical calculations of the electron properties of RuLp, RuLm, RuLo, and BX molecules (see Figure 1), as well as their stochastic modeling on the surface of a TiO_2_ semiconductor.

The hexafluorophosphate (PF_6_^−^) environment stabilized the studied organic molecules. Two PF_6_^−^ molecules were added to each of the RuLp, RuLm, and RuLo molecules, and four to each of the BX molecules. The geometries of the organic complexes were modeled in a vacuum, applying the DFT/B3LYP method augmented by the second-order scalar relativistic effects within the Douglas–Kroll–Hess (DKH2) formalism [34,35,36] using the jorge-TZP-DKH basis set [37]. In our previous work, we reported that the geometry of the tris(bipyridine)ruthenium(II)-based molecules optimized by DFT/B3LYP-DKH2 (jorge-TZP-DKH) could reproduce the experimental results [38]. The molecular structures were optimized, with a gradient convergence tolerance of less than 10^−5^ Hartree/Bohr at a restricted Hartree–Fock (RHF) level. Equilibrated geometries of the molecules were found by applying the quadratic approximation (QA) optimization algorithm based on the augmented Hessian technique, where at the end of the geometry search, the Hessian evaluation was performed to exclude structures, giving the negative modes and ensuring the thermodynamic equilibrium of the molecule. The calculations were performed in the Gaussian 16 program package.

The electron properties of the studied molecules were also calculated for their optimized structures using the DFT/B3LYP-DKH2(jorge-TZP-DKH) formalism. The RHF energy convergence criterion was chosen to be 10^−12^ Hartree. The UV–Vis absorption spectra were calculated using the iterative Davidson algorithm with an accuracy of 10^−12^ Hartree.

The (111) surface of the TiO_2_ in anatase form was constructed, and its energy was optimized in the Materials Studio program using the Forcite simulation module, implementing the Universal force field [39]. The convergence criterion was chosen as 10^−4^ kcal/mol for the energy convergence and 10^−3^ kcal/(molÅ) for the forces. In consequence, the orthorhombic unit cell, with its edges equal to 37.94 Å × 21.36 Å × 66.68 Å, including the TiO_2_ (111) surface, was built.

To find the most stable adsorption sites of the organic molecules on the anatase TiO_2_ (111) surface, Monte Carlo (MC) simulations were performed. To characterize the electrostatic and VdW interactions between the organic molecules and semiconductor, the atom charges were taken from the quantum chemical calculations performed for the organic molecules via the procedures described in the previous paragraph and the calculations performed for the semiconductor in the Vienna ab initio simulation package (VASP) in version vasp.5.4.4 (VASP Software GmbH, Vienna, Austria) [40,41,42]. The quantum chemical calculations were performed with generalized gradient approximation (GGA) applied to the density functional theory (DFT/GGA) method using the PBE functional [43]. The electron structures of all atoms were described by sv-GW basis sets. Computations were augmented by the Hubbard correction to avoid problems occurring with a description of the 3d electrons [44]. In this case, the rotation-invariant LSDA + U method introduced by Liechtenstein et al. was used [45]. The U and J parameters representing the Coulomb interaction energy and exchange energy equal to U_Ti_ = 9.25 eV and J_Ti_ = 1.00 eV, respectively, were specified separately [46].

Building the composite system, three RuLp, or three RuLm, or three RuLo, or two B1, or two B2, or two B3 molecules were located in the simulated unit cell. In this case, the simulated annealing of the adsorption locator method implemented in Materials Studio (version: MS 8.0) was used. Automated temperature control checking was selected for fifty cycles. The geometry of the system in each cycle was optimized to find the most stable structure of the created hybrid system. All the used computational parameters were the same as the ones applied to perform geometry optimization of the TiO_2_ surface. The simulations were performed using the Universal force field [39].

The structures with the lowest total energy were taken to calculate the environmental influence on the electron and optical properties of the RuLp, B1, B2, and B3 molecules. In this case, the discreet local field model described in our previous work was used [47], taking into consideration the intermolecular interaction occurring between the TiO_2_ structure and Ru-based molecules. In discrete local field theory, the local fields are computed by considering the molecular environment rigorously, without resorting to a continuum or mean-field approximations.

### 4.2. Experimental Setup and Sample Preparation

The syntheses of ruthenium compounds RuLp, B1, B2, and B3 are described in our previous works [22,23,48]. UV–vis absorption spectra were obtained according to a previously applied procedure [23] using 10^−5^ M acetonitrile (Honeywell) solutions of investigated dyes on a Shimadzu UV–3600 Plus UV–Vis–NIR (Shimadzu Corporation, Kioto, Japan) spectrophotometer in a 1 cm path length quartz cell.

Optical absorption measurements of solid samples were carried out in the glass/FTO/TiO_2_/Ru dye configuration using a Perkin-Elmer Lambda 10 spectrophotometer. The procedure for the formation of samples is described in the reference [47]. A 1.5 μm thick TiO_2_ layer was deposited on an FTO-covered glass substrate by spinning at 1000 rpm. Layer thicknesses were determined using a Tencor Alpha Step 500 profiler (KLA Corporation, Milpitas, CA, USA).

## 5. Conclusions

The interaction between dye molecules and semiconductor surfaces in DSSCs is a complex but crucial aspect that significantly influences the efficiency of solar cells. Understanding the mechanisms of dye adsorption, electron transfer, and the energetic alignment of molecular orbitals with the semiconductor conduction band is essential for the development of high-performance DSSCs.

The energetics of the dye–TiO_2_ interaction can be analyzed through molecular mechanics calculations. Studies utilizing Monte Carlo simulation have shown that the binding energy between dye molecules and TiO_2_ is significantly influenced by the molecular configuration of the dye, especially by the position of the anchoring group. For instance, it has been observed that dyes tend to adopt a horizontal orientation on the TiO_2_ surface, which can enhance the overlap between the dye’s molecular orbitals and the conduction band of TiO_2_. This orientation is critical, as it can lead to more efficient electron transfer processes. Our findings show that the position of the anchor groups significantly affects the bonding interactions with the TiO_2_ surface, which in turn influences charge transfer efficiency.

In conclusion, the results presented highlight the critical role of adsorption configurations and the interaction strength between the studied ruthenium-based dyes and the TiO_2_ surface. Notably, the RuLp dye demonstrates the strongest interaction with the TiO_2_ surface, with a clear preference for a perpendicular alignment of its dipole moment. This leads to stronger adsorption after the removal of a molecule, making RuLp more likely to form a monolayer on the surface. The RuLm and RuLo molecules interact less significantly with the TiO_2_ surface. The differences in adsorption behaviors occurring between mononuclear dyes are attributed to the structural characteristics of the dyes, such as the positioning of anchor groups (-COOH). The RuLp molecules tend to be strongly connected to the surface of the semiconductor, but the RuLo and RuLm molecules are only physisorbed on the TiO_2_ surface.

The binuclear dyes prefer a bidentate connection with TiO_2_. It was shown that the increase in the number of anchoring groups in the molecule structure increases its ability to bond with the semiconductor, which could affect its performance in dye-sensitized solar cells. The findings are consistent with previous experimental studies, reinforcing the importance of anchoring groups in enhancing adsorption capacity and improving electron transfer in DSSC applications.

Furthermore, the UV–vis spectral analysis shows that the adsorption of the dyes on TiO_2_ influenced the absorption properties, with subtle shifts in the MLCT bands. These results emphasize the critical nature of dye–semiconductor interactions for optimizing the performance of DSSCs. The combination of experimental and computational approaches provides a comprehensive understanding of the adsorption behavior, which is essential for the design of more efficient dyes for solar energy conversion applications. Continued research into optimizing dye structures, enhancing surface interactions, and addressing stability issues will be vital for advancing the commercial viability of dye-sensitized solar technology. As the demand for renewable energy sources grows, DSSCs hold the potential to play a significant role in the future of solar energy conversion.

## Figures and Tables

**Table 1:** The total energy of the investigated dyes adsorbed on the TiO_2_ (111) surface, dyes’ adsorption energy, and release energy calculated using the Monte Carlo method.

Molecule	Total Energy [kcal/mol]	Total Energy/Atom [kcal/mol]	Adsorption Energy [kcal/mol]	$\partial E_{ad}/\partial N_{i}$ [kcal/mol]
RuLp	3.98 × 10^3^	0.45 × 10^3^	−113.55	−52.24
RuLm	6.95 × 10^3^	0.78 × 10^3^	−109.56	−42.50
RuLo	6.90 × 10^3^	0.77 × 10^3^	−83.04	−33.47
B1	5.29 × 10^3^	0.55 × 10^3^	−72.72	−43.64
B2	5.05 × 10^3^	0.53 × 10^3^	−68.77	−53.71
B2_H_	5.02 × 10^3^	0.52 × 10^3^	−83.36	−53.94
B3	5.45 × 10^3^	0.55 × 10^3^	−84.11	−52.61
B3_H_	5.44 × 10^3^	0.55 × 10^3^	−81.34	−46.84

**Table 2:** The distance between adsorbed molecules and the TiO_2_ (111) surface measured along a possible bond between oxygen with a broken hydrogen bond and the nearest Ti atom, modeled using the Monte Carlo method.

Molecule	RuLp	RuLm	RuLo	B1	B2	B2_H_	B3	B3_H_
Distance [Å]	1.05	2.13	2.42	1.35	1.15	2.39	1.08	1.47

## Data Availability

Computationally obtained data available from K.F-S. and M.M-J on personal request. The experimental data are available from D.P. and M.Z. on personal request.
